# Supplementary material for: The Oxysterol 25-Hydroxycholesterol Inhibits Replication of Murine Norovirus
Source: Viruses. 2019 Jan 24;11(2):97. doi: 10.3390/v11020097 (PMC6409565; doi:10.3390/v11020097)
Supplement: Supplementary file 1 [file viruses-11-00097-s001.pdf]

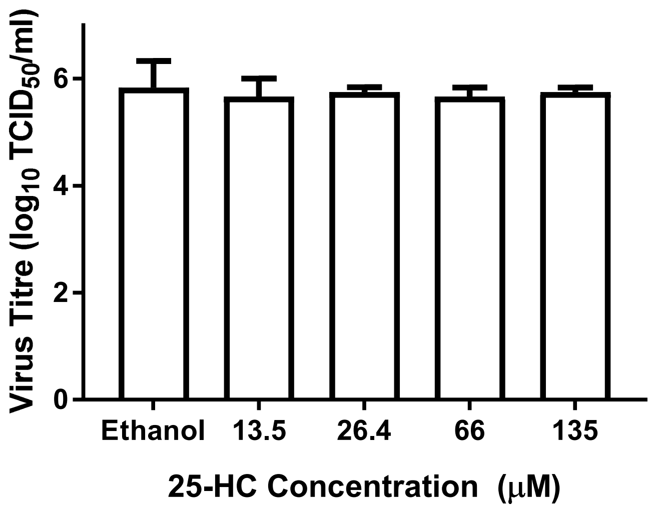

Figure S1. The effect of 25-HC on the viral capsid. Unpurified stocks of MNV were incubated with 25-HC at a range of concentrations or ethanol-only control at 4 h at 37 °C before dilution and measurement of infectivity by $TCID_{50}$ assay (n=2 ± SEM).
